# The Effect of Perceived Supportive Resources on Counterproductive Work Behaviors: The Mediating Role of Thriving at Work Among Nurses

**DOI:** 10.3390/healthcare14162564

**Published:** 2026-08-16

**Authors:** Hamza Moafa

**Affiliations:** Community and Psychiatric Mental Health Nursing Department, College of Nursing, King Saud University, P.O. Box 642, Riyadh 11421, Saudi Arabia; hmoafa@ksu.edu.sa

**Keywords:** perceived supportive resources, thriving at work, counterproductive work behaviors, nurses, Saudi Arabia

## Abstract

**Highlights:**

**What are the main findings?**

- Among nurses in Saudi Arabia, individual, work, and interpersonal work resources were each negatively related to counterproductive work behaviors and positively related to thriving at work.
- The associations between each of the three resource domains and counterproductive work behaviors were fully accounted for by indirect paths through thriving at work, with the largest indirect effect observed for work resources.

**What are the implications of the main findings?**

- Healthcare organizations seeking to reduce counterproductive work behaviors may consider strengthening nurses’ individual, work, and interpersonal resources, and cultivating workplace conditions that foster thriving.
- Given that supportive resources appear to be associated with behavior through thriving at work, interventions targeting fulfillment, meaning, and joy at work may enhance the effectiveness of broader organizational strategies for the nursing workforce.

**Abstract:**

**Background:** Counterproductive work behaviors (CWBs) are voluntary employee behaviors that harm the organization or its members, ranging from minor incivility and withdrawal to overt acts of misconduct. CWBs among nurses are widespread and carry adverse consequences for patients, nurses, and healthcare organizations, often reflecting problematic working conditions rather than individual character flaws. Perceived supportive resources, comprising individual resources (personal strengths and overall health), work resources (staffing and professional development), and interpersonal work resources (supervisor support and participation in unit decisions), have been proposed as protective factors. Thriving at work has been proposed as a potential protective motivational state that may account for how these resources are associated with behavior. **Aim:** This study examines how individual resources, work resources, and interpersonal work resources are associated with CWBs, and whether thriving at work mediates these associations among nurses in Saudi Arabia. **Methods:** One hundred twenty-nine nurses participated in this cross-sectional study between February and March 2026. Data were collected using the Thriving in Nursing Questionnaire (THINQ) and the Individual Work Performance Questionnaire (IWPQ). Data were analyzed via correlation, multiple regression, and mediation with bootstrapping. **Results:** Individual resources (r = −0.363), work resources (r = −0.270), and interpersonal work resources (r = −0.338) were each negatively associated with CWBs (all *p* < 0.001), and each was positively associated with thriving (r = 0.699, 0.793, and 0.794, respectively; all *p* < 0.001). Thriving at work was negatively associated with CWBs (r = −0.408, *p* < 0.001), and the associations of individual resources (indirect β = −0.212), work resources (β = −0.416), and interpersonal work resources (β = −0.301) with CWBs were fully accounted for by indirect effects through thriving, with all direct effects nonsignificant and the largest indirect effect for work resources (95% CI [−0.611, −0.217]). **Conclusions:** Thriving at work emerged as a key correlate through which individual, work, and interpersonal work resources were linked to CWBs among nurses. Enhancing thriving may help reduce CWBs, though longitudinal research is needed to confirm directionality.

## 1. Introduction

Counterproductive work behaviors (CWBs), a form of negative workplace conduct, represent a serious occupational concern, particularly prevalent in healthcare settings. They exist on a spectrum, ranging from minor incivility and withdrawal to overt acts of misconduct and deviance, leading to psychological distress and diminished organizational functioning [1,2,3,4,5]. Nursing’s hierarchical structure and emotional demands make the profession particularly vulnerable to such behaviors [6,7]. The negative consequences of CWBs include physical, psychological, financial, and organizational consequences for patients, nurses, and healthcare institutions [8]. Through organizational socialization, healthcare institutions help nurses adjust to their roles, adopt workplace values, and develop job-related skills [9]. Despite these efforts, CWBs persist among nurses [6,7] and largely reflect problematic working conditions rather than individual character flaws [10,11]. Healthcare organizations therefore have the responsibility of creating healthier work environments that prevent such behaviors. This concern is particularly relevant in Saudi Arabia, where the healthcare system is undergoing rapid transformation under Vision 2030 and the National Transformation Program [12], creating distinct organizational dynamics that warrant context-specific investigation.

Thriving at work describes a psychological state in which employees experience personal well-being, advance professionally, feel valued by others, and remain purposefully connected to an organization whose success is shared at every level [13]. Conceptualized as a motivational state, thriving at work is thought to buffer employees against the detrimental effects of workplace stressors. Meta-analytic evidence associates thriving at work with increased commitment, more favorable job attitudes, and better performance [14], and organizational conditions such as workplace culture have been associated with thriving at work among nurses [15]. Beyond these direct associations, a growing body of research identifies thriving at work as a mediating mechanism in stressful work environments [16,17,18]. However, the mediating role of thriving at work in the pathway linking perceived supportive resources to CWBs among nurses remains poorly understood.

Counterproductive work behaviors among nurses have been linked to a range of antecedents, including destructive leadership, organizational culture, interpersonal conflict, work-related coping strategies, occupational stress, job demands, moral disengagement, ethical climate, and ostracism [11,19,20]; work ethics has also been linked to CWBs among nurses, with workplace ostracism playing a mediating role in this relationship [21]. Among attitudinal mediators, perceived organizational justice has been reported to mediate the association between organizational politics and CWBs [22], while organizational identification and commitment have been identified as mediators [23,24]. Organizational cynicism has been reported to link stressors such as job insecurity to CWBs [25], and engagement, workplace loneliness, and employee silence have been identified as additional mediators between job conditions and CWBs [2,26,27]. Similarly, a study [28] reported that organizational commitment and perceived organizational justice mediated the association between Dark Triad personality traits and CWBs among Chinese physicians. Machiavellian leadership has been associated with CWBs through perceived abusive supervision [29], and abusive supervision has been linked to CWBs through emotional exhaustion and defensive responses [1,30]. Workplace stressors such as work-related identity discrepancy, job insecurity, and sleep impairment have been linked to CWBs through statistical mediators including emotional exhaustion, job stress, and organizational identification [4,24,31], suggesting that counterproductive behaviors function as responses to resource depletion, consistent with conservation of resources (COR) theory [32,33].

While the foregoing literature clarifies how negative conditions relate to CWBs, far less attention has been paid to the protective resources and motivational states that may buffer against them. A prior study [15] found that organizational culture was positively associated with nurses’ thriving at work, with affective commitment and work engagement partially mediating this relationship. A smaller but growing body of work has examined thriving itself as a mediator. In a two-study investigation conducted in China and Australia, Jiang et al. [16] found that thriving mediated the relationships of task identity and autonomy with job satisfaction. Shen et al. [17], studying 308 Chinese clinical nurses, reported that thriving mediated the link between psychological resilience and work performance. Drawing on a national sample of 409 Swedish telephone nurses, Engström et al. [18] demonstrated that thriving mediated the associations between structural empowerment and a range of outcomes, namely work–personal life benefits, job performance, work-related stress symptoms, and turnover intentions.

The present study integrates several complementary frameworks into a single rationale. The job demands–resources (JD-R) model [34,35,36] provides the organizing framework: perceived resources are linked to positive behavioral outcomes through a motivational pathway, and in this study thriving at work represents that motivational state. COR theory [32] complements the JD-R model by linking resource availability to behavioral outcomes: when resources are depleted, counterproductive behaviors become more likely as responses to resource depletion [33]. The specific resource domains examined here are drawn from Arnetz et al. [37], whose model of thriving in the nursing workforce proposes that perceived supportive resources across individual, work, and interpersonal domains are independently associated with thriving at work; this model also supplies the study’s operationalization of thriving as fulfillment, meaning, and joy at work, consistent with the National Academy of Medicine’s National Plan for Health Workforce Well-Being [38], which identifies these states as foundations of thriving and as prerequisites for patient safety and care quality. For the interpersonal domain specifically, social exchange theory [39] adds a relational dimension, proposing that employees who perceive supportive and fair treatment reciprocate with positive rather than harmful behaviors. Convergent support comes from self-determination theory, which specifies the psychological needs, such as autonomy, that workplace resources are expected to satisfy in relation to sustained motivation [40], and from Kanter’s structural empowerment theory, which links staff well-being and effectiveness to access to information, support, resources, development opportunities, and formal and informal power, all of which are shaped by workplace relationships and organizational roles [41,42].

Taken together, these frameworks converge on three expectations: each resource domain should be positively associated with thriving at work; thriving should be negatively associated with CWBs; and thriving should therefore account for the associations between resources and CWBs.

**Figure 1:**
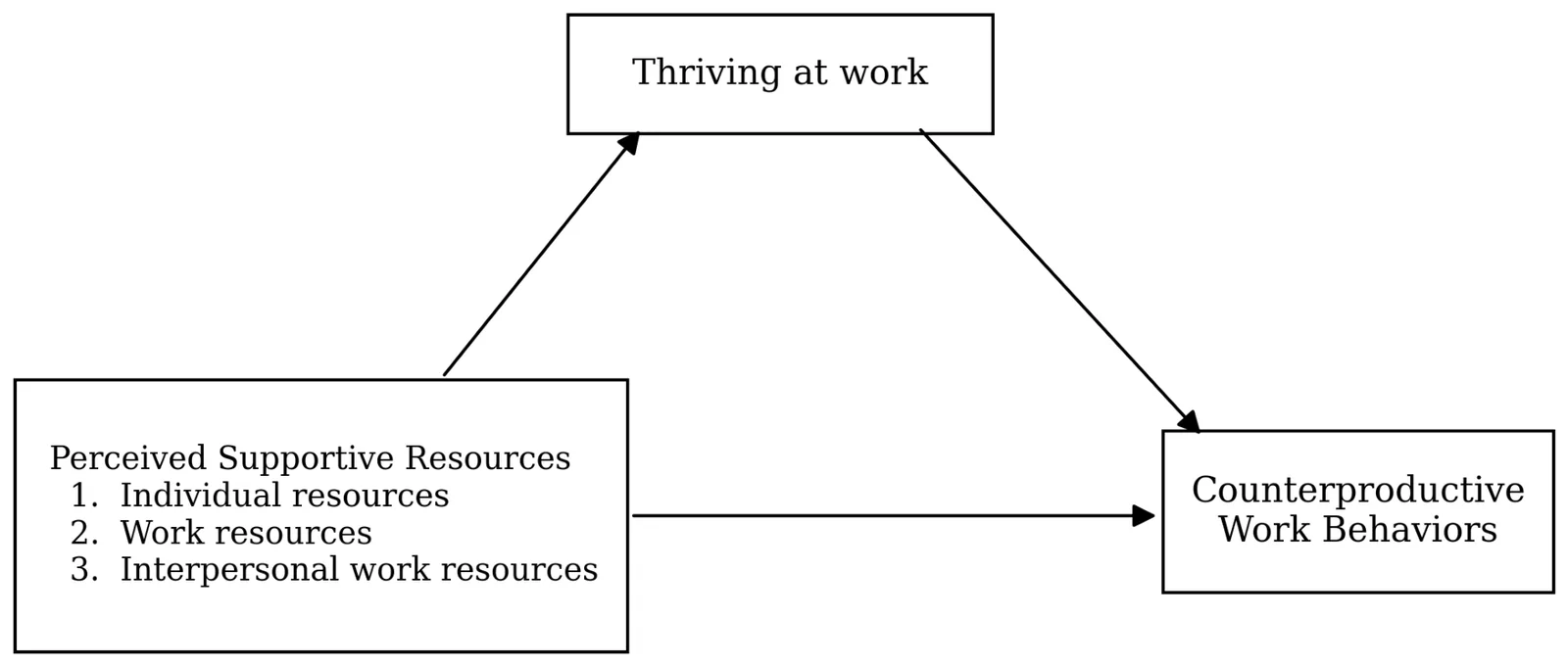
The study framework examining the mediating role of thriving at work between perceived supportive resources and counterproductive work behaviors.

Although prior research has examined the determinants of CWBs and the benefits of thriving at work separately, the role of perceived supportive resources in fostering thriving and, in turn, the relationship with CWBs among nurses remains underexplored, particularly in the Saudi Arabian context, where the multinational nursing workforce and evolving healthcare system present unique organizational conditions. Integrating the JD-R model [34,35,36], COR theory [32], social exchange theory [39], and the Arnetz et al. thriving model [37], this study addresses this gap by examining (1) the relationship between perceived supportive resources and CWBs and (2) whether thriving at work mediates this association among nurses working in Saudi Arabia. The conceptual model underpinning these analyses is depicted in Figure 1. Given the cross-sectional design, the analyses were not intended to test causal mediation, temporal precedence, or directional mechanisms.

## 2. Materials and Methods

### 2.1. Study Design

A cross-sectional, correlational design was adopted, as it is well suited to examining the associations among perceived supportive resources, thriving at work, and CWBs at a single point in time. Validated psychometric instruments were used for data collection, and the statistical procedures applied were appropriate for mediation modeling and inferential interpretation.

### 2.2. Study Setting and Participants

Data were collected from nurses nationwide across Saudi Arabia through an online survey administered via Qualtrics (Provo, UT, USA). Participants were recruited using a non-probability strategy that combined convenience and snowball sampling. Eligibility was restricted to nurses currently employed in healthcare settings within Saudi Arabia. Simulation-based work employing the percentile bootstrap method indicates that a sample of approximately 90 participants provides adequate power (0.80) to detect a medium-sized indirect effect in a simple mediation model at α = 0.05 [43]. The final sample (N = 129) exceeded this threshold, providing adequate power to detect medium-sized indirect effects in the simple mediation models tested; the study was not designed or powered to detect small indirect effects or to estimate more complex models involving multiple simultaneous predictors or covariates. Of the 190 responses received, 129 contained complete data on all study measures and were retained for analysis. The remaining 61 responses were excluded because respondents consented to participate but discontinued the survey before completing the study instruments, leaving incomplete data on the primary measures; no other exclusion criteria were applied. Data were collected between 5 February and 11 March 2026.

### 2.3. Study Instruments

This study employed three structured questionnaires to collect data from participants. Two were standardized psychometric instruments, and the third was a researcher-developed demographic questionnaire capturing age, gender, years of experience, and qualifications, as well as nationality, hospital type, department, frontline status, and shift pattern.

#### 2.3.1. Perceived Supportive Resources

The Thriving in Nursing Questionnaire (THINQ), developed and validated by Arnetz et al. [37], was used to assess perceived supportive resources. The instrument is a 15-item self-report measure comprising three independently scored subscales reflecting different domains of perceived supportive resources.

- Work resources (6 items; 0 = completely disagree to 10 = completely agree): This subscale reflects nurses’ appraisal of the work environment, such as staffing adequacy and professional development opportunities.
- Interpersonal work resources (6 items; same 0–10 scale): This subscale captures relational and organizational factors, such as supervisor support and involvement in unit-level decision-making.
- Individual resources (3 items; 0 = very poor to 10 = very good): This subscale captures personal strengths, concentration, and overall health.

Items within each subscale are summed, with higher scores reflecting greater perceived resources. Possible summed scores therefore range from 0 to 60 for work resources, from 0 to 60 for interpersonal work resources, and from 0 to 30 for individual resources. The distinctness of the three resource subscales was supported by factor analysis in the original validation study by Arnetz et al. [37]. Reliability in the original validation was reported as McDonald’s ω = 0.87, 0.83, and 0.88 for individual resources, work resources, and interpersonal work resources, respectively [37]. In the present sample, the subscales demonstrated good internal consistency, with Cronbach’s α = 0.87, 0.88, and 0.85 for individual resources, work resources, and interpersonal work resources, respectively.

#### 2.3.2. Thriving at Work

Thriving at work was assessed with the three-item thriving subscale of the THINQ [37], scored independently of the 15 resource items, capturing fulfillment, meaningfulness, and joy at work. Items were rated from 0 (completely disagree) to 10 (completely agree) and summed into a single thriving score, yielding a possible range of 0 to 30. Development of the questionnaire was conceptually grounded in the National Academy of Medicine’s National Plan for Health Workforce Well-Being [38]. Prior use in registered nurses showed good reliability (Cronbach’s α = 0.87) [44]. In the present sample, principal component analysis supported a unidimensional structure (factor loadings = 0.78–0.88; inter-item correlations = 0.52–0.63), and internal consistency was Cronbach’s α = 0.79.

#### 2.3.3. Counterproductive Work Behaviors

Counterproductive work behaviors were assessed using the Individual Work Performance Questionnaire (IWPQ), a validated instrument developed by Koopmans et al. [45]. Only the counterproductive work behaviors subscale was used, capturing negative workplace behaviors such as complaining about trivial matters and exaggerating problems at work. The subscale comprises five items rated on a 5-point Likert scale ranging from 0 (never) to 4 (often), reflecting the frequency of each behavior over the preceding three months; higher mean scores indicate higher levels of counterproductive behaviors, with possible mean scores ranging from 0 to 4. The subscale demonstrated acceptable internal consistency in the original validation study, with Cronbach’s α = 0.79 [45]. In the present study, the subscale demonstrated comparable reliability, with Cronbach’s α = 0.77.

### 2.4. Data Collection and Research Ethics

Ethical approval for the study was obtained from the Institutional Review Board of King Saud University (IRB reference number KSU-HE-25-1401), in accordance with national regulations governing research involving human participants. Once approval was secured, the survey link was circulated electronically across nurses’ professional and personal networks, and recipients were invited to forward the link to other nurses working in Saudi Arabia, reflecting the snowball component of the sampling strategy. The online survey opened with an electronic information sheet, which set out the study’s objectives, eligibility, voluntary participation, confidentiality, and the right to withdraw without consequence. Every response was anonymized, coded, and stored securely. Participants indicated their informed consent electronically at the beginning of the survey and were required to do so before proceeding to the questionnaire items, in accordance with the IRB-approved protocol.

### 2.5. Statistical Analysis

Participant characteristics and study variables were summarized descriptively: continuous variables were reported as means and standard deviations, and categorical variables as absolute counts with corresponding percentages. Associations among continuous variables were examined using Pearson’s correlation coefficient (r). Prior to the mediation analyses, multicollinearity was assessed via variance inflation factors (VIFs); when the three perceived supportive resource domains were entered simultaneously as predictors of thriving at work and CWBs, all VIFs fell below the threshold of 5. CWBs served as the dependent variable in multiple linear regression models, from which adjusted regression coefficients (β) and their standard errors (SEs) were derived. To evaluate the potential for common method bias, Harman’s single-factor test was applied [46]: all items were subjected to an unrotated exploratory factor analysis, and the first factor explained 41.9% of the variance, beneath the conventional 50% criterion, indicating that common method bias was unlikely to pose a substantial concern in the present data. The mediating role of thriving at work in the association between perceived supportive resources and CWBs was examined using the SPSS PROCESS macro (version 4.2; Model 4), in accordance with Hayes’ guidelines [47]. Separate mediation models were tested for each domain of perceived supportive resources (individual, work, and interpersonal), as the THINQ treats each subscale as an independently scored construct rather than yielding a composite total score [37]. All models were estimated without demographic or work-related covariates; the sample size was powered for simple mediation models rather than models incorporating multiple simultaneous covariates [43], and the rationale for this decision is addressed further in the Limitations. The significance of the indirect effect was tested using a bootstrap procedure with 5000 resamples to generate bias-corrected 95% confidence intervals (CIs). Mediation was indicated when the indirect effect reached significance, that is, when the bootstrap confidence interval excluded zero. In line with contemporary guidance [48], a significant indirect effect combined with a nonsignificant direct effect was interpreted as full mediation, whereas a direct effect that remained significant was interpreted as partial mediation. These labels characterize statistical patterns of association in cross-sectional data and carry no implication of causal mediation. Prior to analysis, all variables were standardized [47]. None of the standardized instruments employed has established cut-off scores, normative values, or reference data for the population studied; all instrument scores were therefore analyzed as continuous variables, and no categorization of participants into score bands was undertaken. Owing to the cross-sectional design, the findings were interpreted as associations rather than causal effects. Two-tailed tests were applied throughout, with statistical significance defined as *p* < 0.05, and all analyses were conducted in IBM SPSS Statistics, version 31.

## 3. Results

### 3.1. Sample Characteristics

A total of 129 nurses participated in the study. Table 1 presents the sociodemographic characteristics of the participants. The sample had a mean age of 32.91 years (SD = 6.97) and an average of 9.34 years of nursing experience (SD = 6.56). The majority were female (n = 90, 69.8%) and held a bachelor’s degree in nursing (n = 98, 76.0%). Participants worked across diverse hospital types and clinical units, and approximately half were assigned to rotating shifts (n = 67, 51.9%). Additional sample characteristics, including nationality, hospital type, and department, are presented in Table 1.

### 3.2. Descriptive Statistics and Correlations

Descriptive statistics and correlations for all major study variables are presented in Table 2. The mean score for thriving at work was 20.66 (SD = 5.56) on a possible range of 0 to 30 (scale midpoint = 15). The CWB subscale showed a mean of 2.36 (SD = 0.82) on a possible mean range of 0 to 4 (scale midpoint = 2). All scales demonstrated good internal consistency, with Cronbach’s α coefficients ranging from 0.77 to 0.88. As shown in Table 2, thriving at work was strongly and positively associated with perceived supportive resources (r = 0.699 to 0.794, all *p* < 0.001), indicating that nurses with higher perceived supportive resources tend to experience greater thriving at work. Additionally, thriving at work showed a significant negative correlation with CWBs (r = −0.408, *p* < 0.001), suggesting that nurses with higher thriving report fewer counterproductive behaviors. Regarding the predictor–outcome associations, CWBs were significantly and negatively correlated with each domain of perceived supportive resources: individual resources (r = −0.363, *p* < 0.001), work resources (r = −0.270, *p* < 0.001), and interpersonal work resources (r = −0.338, *p* < 0.001). These results indicate that nurses reporting more perceived supportive resources also report higher thriving and lower CWBs.

### 3.3. Mediation Analyses: Thriving at Work as a Mediator

**Figure 2:**
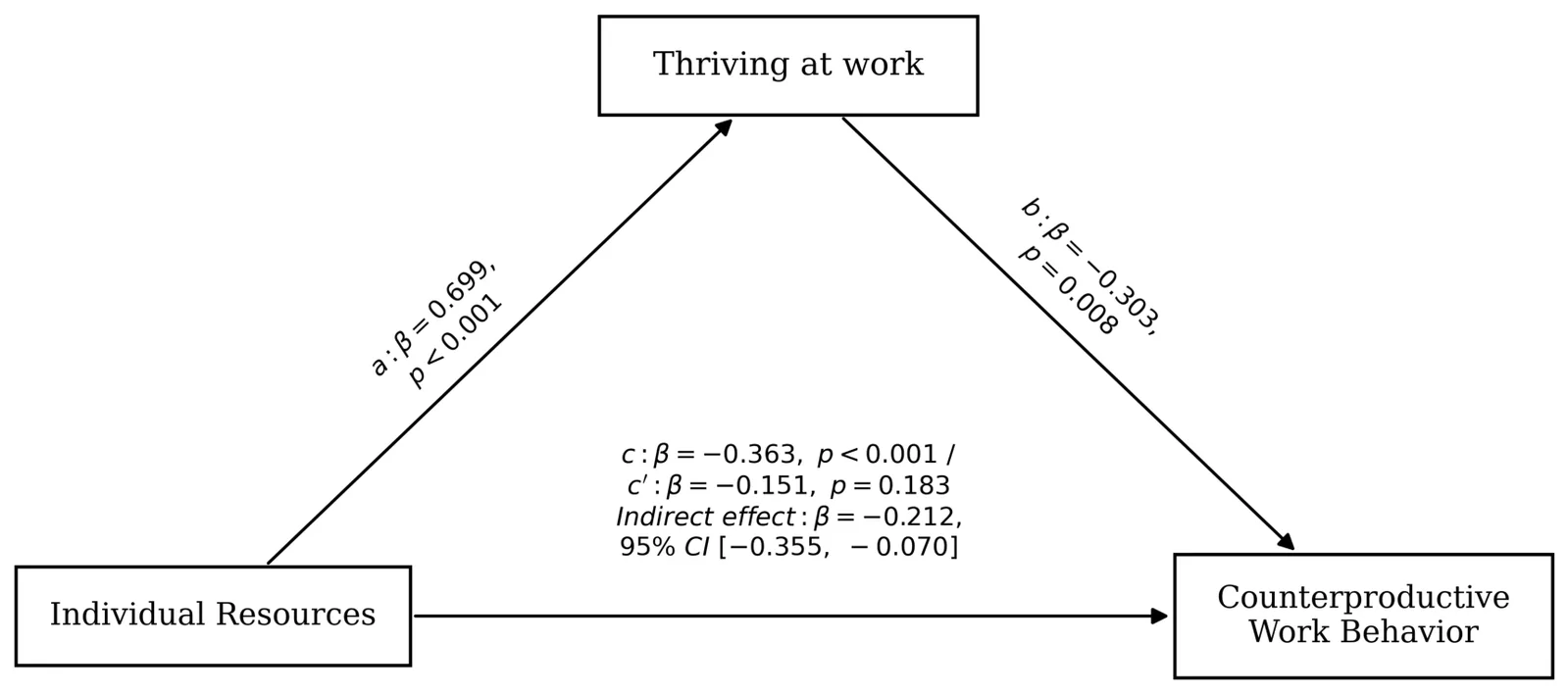
Mediation Model 1: Individual resources → Thriving at work → Counterproductive work behaviors.

A series of separate mediation analyses was conducted to determine whether thriving at work carried the association between each domain of perceived supportive resources and CWBs, with thriving specified as the mediator in every model. Direct and indirect effects were estimated through bias-corrected bootstrapping, drawing on 5000 resamples to construct 95% confidence intervals. Thriving at work fully accounted for the association between individual resources and CWBs. In Model 1, individual resources were significantly associated with thriving at work (β = 0.699, *p* < 0.001), and thriving was in turn negatively associated with CWBs (β = −0.303, *p* = 0.008). Although the total effect was significant (β = −0.363, *p* < 0.001), the direct effect was not (β = −0.151, *p* = 0.183); the indirect effect, by contrast, was significant, with a bootstrap confidence interval that did not span zero (indirect effect = −0.212, 95% CI [−0.355, −0.070]) (Figure 2; Table 3).

**Figure 3:**
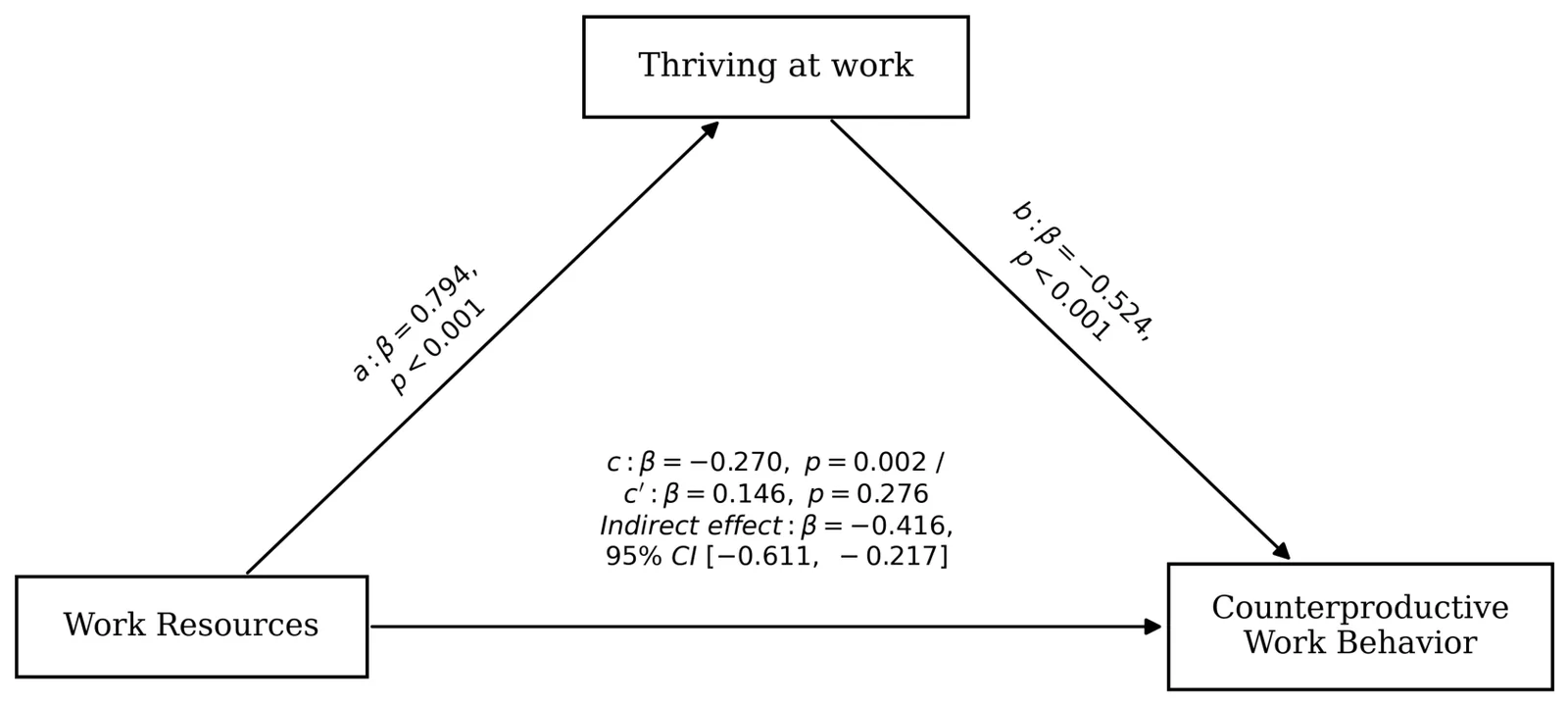
Mediation Model 2: Work resources → Thriving at work → Counterproductive work behaviors.

Work resources followed a comparable pattern. In Model 2, work resources were significantly associated with thriving at work (β = 0.794, *p* < 0.001), which was negatively associated with CWBs (β = −0.524, *p* < 0.001). The total effect was significant (β = −0.270, *p* = 0.002), whereas the direct effect was not (β = 0.146, *p* = 0.276); the indirect effect remained significant, its bootstrap confidence interval excluding zero (indirect effect = −0.416, 95% CI [−0.611, −0.217]) (Figure 3; Table 3).

**Figure 4:**
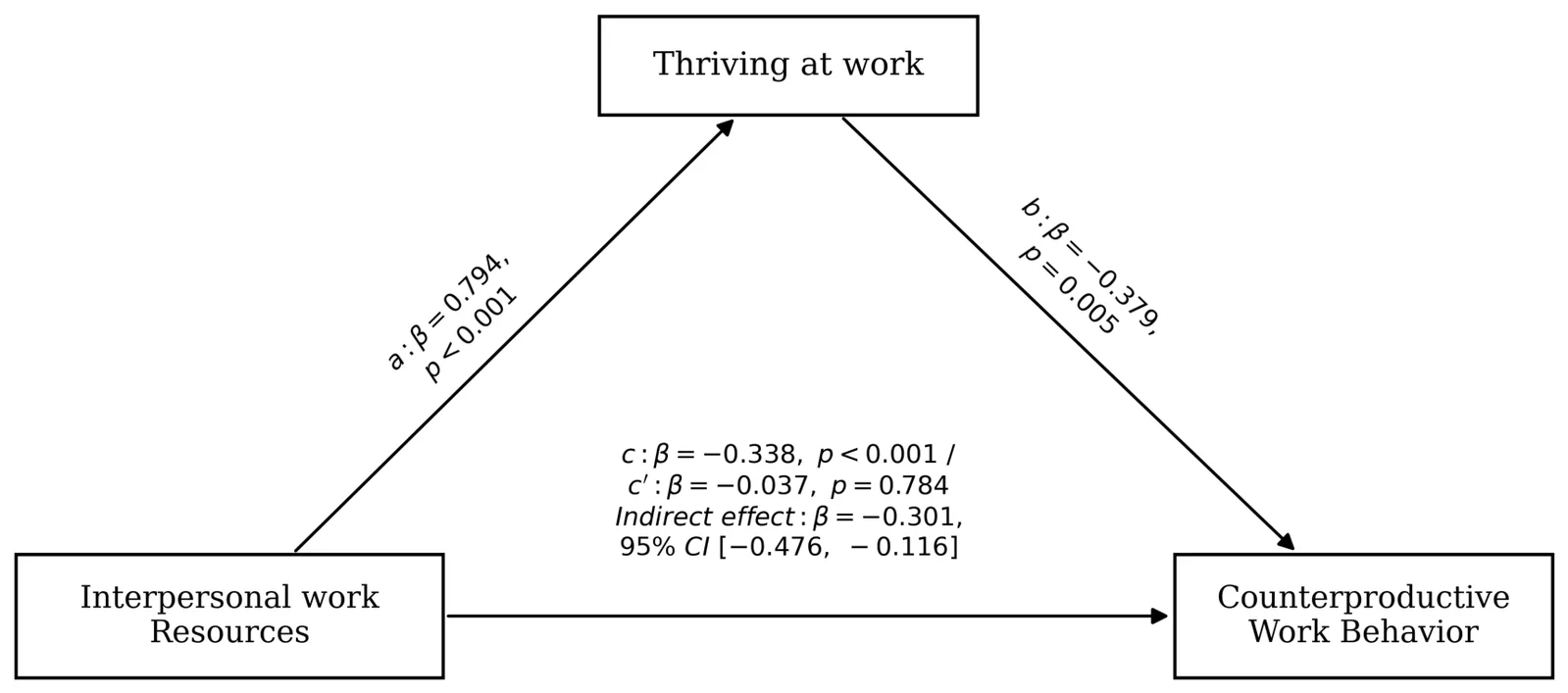
Mediation Model 3: Interpersonal work resources → Thriving at work → Counterproductive work behaviors.

Interpersonal work resources replicated this pattern. In Model 3, they were significantly associated with thriving at work (β = 0.794, *p* < 0.001), which in turn was negatively associated with CWBs (β = −0.379, *p* = 0.005). The total effect was significant (β = −0.338, *p* < 0.001), the direct effect was not (β = −0.037, *p* = 0.784), and the indirect effect was significant, with a bootstrap confidence interval that did not include zero (indirect effect = −0.301, 95% CI [−0.476, −0.116]) (Figure 4; Table 3).

These findings indicate that the association between each domain of perceived supportive resources and CWBs was fully accounted for by the indirect path through thriving at work, with the strongest indirect pathway observed for work resources.

## 4. Discussion

This study investigated the relationship between perceived supportive resources and CWBs among nurses in Saudi Arabia, and examined the mediating role of thriving at work. The findings indicate that perceived supportive resources across the individual, work, and interpersonal domains were positively associated with thriving at work and negatively associated with CWBs, and that each of these associations was fully accounted for by the indirect path through thriving at work. Collectively, these results emphasize the central role of thriving at work in linking perceived supportive resources to CWBs.

### 4.1. Key Findings of the Study

Four key findings emerged from this study, each consistent with the existing international literature and of particular relevance to the workforce well-being of nurses. First, thriving at work proved to be a significant motivational state, as it was positively associated with perceived supportive resources (r = 0.699 to 0.794, all *p* < 0.001) and negatively associated with CWBs (r = −0.408, *p* < 0.001), supporting its role in workplace functioning. As described by Kleine et al. [14], employees who thrive at work tend to report stronger commitment, more positive job attitudes, and better work performance. Second, perceived supportive resources across all three domains were negatively associated with CWBs. Nurses with greater individual, work, and interpersonal resources reported lower levels of counterproductive behavior, consistent with prior research on the role of job resources in workplace functioning [26,33]. Third, the association between each domain of perceived supportive resources and CWBs was fully accounted for by the indirect path through thriving at work. This pattern is consistent with the JD-R model [34,35,36], which posits that resources are associated with positive outcomes through a motivational pathway. Finally, this pattern highlights that the association between supportive resources and CWBs was accounted for through the motivational state of thriving rather than directly [13,38].

### 4.2. Pattern of Associations Across the Three Models

In Model 2, the direct association between work resources and CWBs reversed in sign once thriving at work was included (c′ = 0.146, *p* = 0.276), although it did not reach statistical significance. This pattern, in which the total and indirect associations are negative while the residual direct path is slightly positive, is consistent with an indirect-association pattern in which thriving at work carries the relationship between work resources and CWBs. The substantive conclusion, that thriving is the resource-related construct most closely associated with CWBs, is unchanged across the three models.

### 4.3. Thriving at Work and Perceived Supportive Resources

Nurses who reported more supportive interpersonal relationships and a more supportive work environment also reported higher thriving at work and lower CWBs. This finding aligns with a recent systematic review by Bergmans et al. [49], in which organizational support and interpersonal support emerged as the most frequently reported antecedents of job satisfaction, well-being, positive mental health, and resilience. While that review focused on forensic healthcare workers, this study adds evidence from Saudi Arabia’s distinctive nursing workforce, which is predominantly expatriate (62.8% non-Saudi in the present sample). The findings thus reflect nurses working within the Saudi healthcare system rather than Saudi nationals or Middle Eastern nurses broadly, a mixed-nationality composition characteristic of the region and relevant to organizational and workforce research. These findings align with self-determination theory [40], which proposes that motivation and personal growth depend on satisfaction of three fundamental needs: autonomy, competence, and relatedness, reflecting social connection. Contexts that support the satisfaction of these needs may be associated with vitality and sustained involvement.

### 4.4. Associations Between Perceived Supportive Resources and CWBs

The negative association between individual resources and CWBs is consistent with theoretical accounts that emphasize the essential role of individual strengths in stressful work environments. According to COR theory [32], lower vitality may reduce the personal resources available for self-regulation, a pattern interpreted as resource depletion preceding counterproductive behavior [33]; depleted energy and concentration have similarly been linked to counterproductive impulses through reduced self-regulatory capacity [50]. The study found a negative association between perceived work resources and CWBs. This finding aligns with the work of Zhao et al. [26], who reported that job resources were negatively associated with counterproductive behaviors, whereas job stressors were positively associated with such behaviors. Negative perceptions of the workplace have been associated with deviant behavior in prior research [51]. Building on the stressor-emotion model of counterproductive behaviors, prior research [52] reported that job plateauing, where employees perceive a lack of professional growth, was associated with counterproductive behaviors, with negative emotions identified as a statistical mediator in cross-sectional data. Interpersonal work resources were also negatively associated with CWBs. This finding aligns with social exchange theory, which offers a relational account of these associations [39]. According to this theory, the way employees are treated within an organization is associated with their behavior: positive interactions are related to constructive performance, whereas unfair treatment is related to detrimental behaviors [39]. Deviant workplace behavior is significantly associated with workplace culture [53]. Such patterns are especially evident when there is a lack of congruence between employees and their organizational environment, alongside a diminished perception of psychological safety [54]. Thriving at work was negatively associated with CWBs in the present study. As defined earlier, thriving at work encompasses personal well-being, professional growth, social appreciation, and purposeful organizational connection [13]. A meta-analysis indicated that employees who thrive tend to report greater organizational commitment, more positive perceptions of their work, and better performance outcomes [14]. According to Sirbu et al. [55], meaningful work has been associated with lower counterproductive behaviors and more positive job attitudes; these patterns have been interpreted in prior literature as reflecting a deterrent function.

### 4.5. Implications for Practice

The findings of the present study have important implications for understanding the role of perceived supportive resources in the nursing workforce. From a practical perspective, supportive workplace environments were associated with higher levels of thriving at work, which was associated with lower CWBs. Accordingly, healthcare organizations may consider initiatives aimed at improving the nursing work environment and nurses’ overall well-being. Organizations may benefit from attending to the quality of workplace relationships and the dynamics of mutual support within healthcare teams. Nurse leaders can foster positive work environments by valuing nurses, providing timely support, and involving them in decision-making to help build a thriving workforce. Specific strategies aligned with each resource domain of the THINQ may strengthen these efforts. For individual resources, organizations may invest in wellness programs that promote nurses’ physical health, concentration, and personal strengths, such as stress management training and adequate rest periods between shifts. For work resources, ensuring adequate staffing levels and providing meaningful professional development opportunities may foster perceptions of a supportive work environment conducive to professional growth. For interpersonal work resources, strengthening supervisor support through leadership development programs and increasing nurses’ participation in unit-level decision-making may enhance the relational aspects of the work environment that were associated with thriving in this study. Although supporting nurses at all levels remains essential, nurse leaders and managers should recognize that support alone may not directly reduce CWBs. Thus, interventions should be designed to enhance nurses’ thriving as a means of strengthening the effectiveness of organizational strategies, though longitudinal intervention research is needed to confirm these associations and clarify their direction. Such interventions could be evaluated through pre–post designs assessing perceived supportive resources and thriving with the THINQ alongside CWBs at follow-up, or through multi-wave studies testing whether changes in resources and thriving precede changes in CWBs over time.

### 4.6. Strengths

A methodological strength of this study lies in the development of a conceptual model that integrates established theoretical frameworks with the existing literature. The study moves beyond the limitation of relying on a single theory that may only capture a partial view of how perceived supportive resources and thriving at work relate to CWBs. Another key strength is the use of bootstrapped indirect-effect analyses, which clarify how the overall associations between each domain of perceived supportive resources and CWBs break down into direct and indirect components through thriving at work. Although these analyses cannot support causal-mediation claims in a cross-sectional design, they provide a more detailed view of the relationships among these constructs than bivariate correlations alone.

### 4.7. Limitations and Future Directions

This study has several limitations. First, convenience and snowball sampling may have introduced selection bias and limited sample diversity; recruitment proceeded through informal professional and personal networks, so nurses with stronger network engagement may be over-represented. Second, the cross-sectional, correlational design precludes causal inference: with all variables measured at a single time point, neither temporal precedence nor the direction of the observed associations can be established. Given that cross-sectional indirect-association estimates can diverge substantially from longitudinal mediation estimates, the bootstrapped estimates produced by the PROCESS analyses should be interpreted as patterns of association consistent with the proposed conceptual model rather than as evidence of causal mechanisms, mediation, or directional pathways. Third, reliance on self-reported questionnaires may have introduced response bias; social desirability is a particular concern for the assessment of CWBs, which respondents may underreport. Fourth, the mediation models were estimated without demographic or work-related covariates (e.g., age, years of experience, workload, organizational factors). The study was designed and powered to test simple mediation models rather than models incorporating multiple simultaneous covariates, so covariate-adjusted analyses were beyond the scope of the present design. This choice is also consistent with methodological guidance cautioning against the atheoretical inclusion of control variables, which can remove substantive variance and bias parameter estimates [56]. Residual confounding by these variables cannot be entirely ruled out, and covariate-adjusted models in larger samples represent a useful direction for future research. Other unmeasured factors, such as personality traits and organizational justice, may likewise offer alternative explanations. Fifth, the instruments employed do not have established cut-off scores or normative reference data for the population studied, so the observed scores cannot be interpreted against external benchmarks and the findings should be understood in relative rather than diagnostic or normative terms. Finally, the sample comprised only nurses employed in healthcare settings in Saudi Arabia, which may limit the generalizability of the findings to nurses in other contexts and healthcare systems; the predominantly multinational nursing workforce, in which internationally recruited nurses constitute a substantial share and face documented challenges related to cultural and organizational adjustment, workload, and retention [12,57,58], was not examined through subgroup comparisons and warrants dedicated investigation. Longitudinal and intervention designs drawing on more diverse, representative samples of nurses across multiple regions and healthcare settings are needed to test whether changes in perceived supportive resources and thriving at work temporally precede changes in CWBs.

## 5. Conclusions

This study provides evidence of a pattern of associations consistent with a conceptual model in which perceived supportive resources across the individual, work, and interpersonal domains are associated with lower CWBs among nurses, with each association fully accounted for by the indirect path through thriving at work. The findings position thriving at work as a key correlate of CWBs in the present sample, with higher levels of thriving associated with lower CWBs. However, the cross-sectional design precludes causal or mediational interpretations, and the bootstrapped indirect-association estimates should be interpreted descriptively rather than as evidence of a mechanism. These findings may inform healthcare organizations’ strategies to strengthen supportive resources and improve work environments, as these conditions were associated with higher thriving at work and lower CWBs among nurses. Future longitudinal and intervention studies should include larger and more diverse samples and further examine how these factors relate to counterproductive behaviors in different healthcare contexts, including whether changes in perceived supportive resources and thriving precede changes in CWBs over time.

## Figures and Tables

**Table 1:** Sociodemographic characteristics of participants (N = 129).

Variable	Category	Mean	SD	n	%
Age (years)		32.91	6.97		
Years of experience		9.34	6.56		
Gender	Male			39	30.2
	Female			90	69.8
Qualification	Diploma in Nursing			17	13.2
	Bachelor’s in Nursing			98	76.0
	Master’s in Nursing			14	10.9
Nationality	Saudi			48	37.2
	Non-Saudi			81	62.8
Hospital	Primary healthcare center			5	3.9
	General hospital			25	19.4
	Specialized hospital			37	28.7
	Academic hospital			21	16.3
	Private hospital or clinic			33	25.6
	Rehabilitation center			3	2.3
	Other			5	3.9
Department	Medical–Surgical			16	12.4
	Emergency			21	16.3
	Intensive Care Unit (ICU)			19	14.7
	Maternity/Obstetric			9	7.0
	Pediatric			11	8.5
	Outpatient Clinic			14	10.9
	Rehabilitation			8	6.2
	Oncology			2	1.6
	Operation Room			5	3.9
	Mental health unit			2	1.6
	Neonatal department			1	0.8
	Isolation			1	0.8
	Dental			1	0.8
	Hemodialysis			1	0.8
	Other			18	14.0
Nurse type	Frontline			89	69.0
	Not frontline			40	31.0
Shift	Morning			57	44.2
	Evening			2	1.6
	Night			3	2.3
	Rotating			67	51.9

**Table 2:** Means, standard deviations, and correlations among study variables (N = 129).

Variable	M	SD	CWBs	Thriving at Work
1. Counterproductive work behaviors	2.36	0.82	—	—
2. Thriving at work	20.66	5.56	−0.408	—
3. Individual resources	20.69	6.36	−0.363	0.699
4. Work resources	45.16	9.33	−0.270	0.793
5. Interpersonal work resources	40.17	10.91	−0.338	0.794

Note. CWBs = counterproductive work behaviors. Possible score ranges: CWBs 0–4 (mean score); thriving at work 0–30; individual resources 0–30; work resources 0–60; interpersonal work resources 0–60. All correlations are significant at *p* < 0.001. — = not applicable.

**Table 3:** Direct and indirect associations between perceived supportive resources, thriving at work, and counterproductive work behaviors (N = 129).

Path	B	SE	β	*p*	Boot SE	95% CI [LL, UL]
Model 1: Individual resources → Thriving at work → CWBs						
Path a (IV → Thriving)	0.612	0.056	0.699	<0.001	—	[0.502, 0.722]
Path b (Thriving → CWBs)	−0.045	0.017	−0.303	0.008	—	[−0.078, −0.012]
Total effect (c)	−0.047	0.011	−0.363	<0.001	—	[−0.068, −0.026]
Direct effect (c′)	−0.020	0.015	−0.151	0.183	—	[−0.048, 0.009]
Indirect effect	—	—	−0.212	—	0.073	[−0.355, −0.070]
Model 2: Work resources → Thriving at work → CWBs						
Path a (IV → Thriving)	0.473	0.032	0.794	<0.001	—	[0.409, 0.537]
Path b (Thriving → CWBs)	−0.077	0.020	−0.524	<0.001	—	[−0.116, −0.038]
Total effect (c)	−0.024	0.008	−0.270	0.002	—	[−0.039, −0.009]
Direct effect (c′)	0.013	0.012	0.146	0.276	—	[−0.010, 0.036]
Indirect effect	—	—	−0.416	—	0.102	[−0.611, −0.217]
Model 3: Interpersonal work resources → Thriving at work → CWBs						
Path a (IV → Thriving)	0.405	0.028	0.794	<0.001	—	[0.351, 0.459]
Path b (Thriving → CWBs)	−0.056	0.020	−0.379	0.005	—	[−0.095, −0.017]
Total effect (c)	−0.025	0.006	−0.338	<0.001	—	[−0.038, −0.013]
Direct effect (c′)	−0.003	0.010	−0.037	0.784	—	[−0.023, 0.017]
Indirect effect	—	—	−0.301	—	0.091	[−0.476, −0.116]

Note. Model 1: R^2^ = 0.49 for the mediator model, F(1, 127) = 121.36, *p* < 0.001; R^2^ = 0.18 for the outcome model, F(2, 126) = 13.67, *p* < 0.001. Model 2: R^2^ = 0.63 for the mediator model, F(1, 127) = 215.88, *p* < 0.001; R^2^ = 0.17 for the outcome model, F(2, 126) = 13.32, *p* < 0.001. Model 3: R^2^ = 0.63 for the mediator model, F(1, 127) = 217.10, *p* < 0.001; R^2^ = 0.17 for the outcome model, F(2, 126) = 12.64, *p* < 0.001. IV = independent variable; CWBs = counterproductive work behaviors. Indirect effect is reported as the completely standardized indirect effect (β). Boot SE = bootstrap standard error based on 5000 bootstrap samples. 95% CI for paths a, b, total effect, and direct effect are ordinary confidence intervals from the PROCESS output. 95% CI for the indirect effect is the bias-corrected bootstrap confidence interval. — = not applicable.

## Data Availability

The data presented in this study are available on request from the corresponding author. The data are not publicly available due to privacy and ethical restrictions.

## Abbreviations

The following abbreviations are used in this manuscript:

CWBs	Counterproductive work behaviors
COR	Conservation of Resources
JD-R	Job Demands–Resources
THINQ	Thriving in Nursing Questionnaire
IWPQ	Individual Work Performance Questionnaire
SPSS	Statistical Package for the Social Sciences
SD	Standard deviation
CI	Confidence interval
VIF	Variance inflation factor
